# mTOR complex 1 signalling regulates the balance between lipid synthesis and oxidation in hypoxia lymphocytes

**DOI:** 10.1042/BSR20160479

**Published:** 2017-02-03

**Authors:** Geng Yin, Yan Liang, Ying Wang, Yuan Yang, Min Yang, Xiao-min Cen, Qi-bing Xie

**Affiliations:** 1 Department of General Medicine, West China Hospital of Sichuan University, Chengdu 610041, China; 2 Department of Rheumatology and Immunology, West China Hospital of Sichuan University, Chengdu 610041, China

**Keywords:** cell apoptosis, cell survival, hypoxia, lymphocyte, mammalian target of rapamycin

## Abstract

Mammalian cells adapt to different environmental conditions and alter cellular metabolic pathways to meet the energy demand for survival. Thus, the metabolic regulation of cells under special conditions, such as hypoxia, should be precisely regulated. During the metabolic regulation, mammalian target of rapamycin (mTOR) plays a vital role in the sensing of extracellular stimulations and regulating intracellular adaptations. Here, we report that mTOR complex 1 (mTORC1) signalling is a central regulator of lipid homoeostasis in lymphocytes. In hypoxia, mTORC1 activity is reduced and shifts lipid synthesis to lipid oxidation. Moreover, knockdown tuberous sclerosis complex 1 (TSC1) constitutively activates mTORC1 activity and impairs the hypoxia-induced metabolic shift. Therefore, TSC1 knockdown enhances hypoxia-induced cell death. Re-inactivation of mTORC1 activity via rapamycin may resist hypoxia-induced cell death in TSC1 knockdown lymphocytes. Our findings provide a deep insight into mTORC1 in the metabolic balance of lipid synthesis and oxidation, and imply that mTORC1 activity should be precisely regulated for the lipid homoeostasis in lymphocytes.

## Introduction

In mammalian cells, energy metabolism needs to be precisely regulated [1]. Under different conditions, cells may rely on different catabolic metabolisms and generate their ATP from various pathways. Therefore, metabolic switches enable cells to adapt to their bioenergetic and biosynthetic needs, respond to changing requirements for survival, expansion and longevity, and match nutrient availability and functional necessities [2]. For example, most mammalian cells produce ATP from oxidative phosphorylation, which is a highly oxygen-dependent manner. However, under some special conditions, such as hypoxia, cells develop multiple mechanisms for adaptation to lower oxygen levels [3]. Cells decrease their mitochondrial oxygen consumption for survival [4]. In the meantime, the lipid metabolism may also change according to the oxygen levels [5]. The anabolic lipid synthesis (lipogenesis) shifts to catabolic lipid oxidation, to meet the energy demand for energy production and cell survival [6]. Thus, the balance of lipid anabolism and catabolism should be tightly regulated and disruption of the adaptative metabolic shift may cause cell death and other diseases.

During the regulation of lipid homoeostasis of mammalian cells, mammalian target of rapamycin (mTOR) signalling is the most important regulator [7]. The mTOR is a serine/threonine kinase that in association with raptor and mLST8 forms a complex (mTOR complex 1 (mTORC1)) and regulates protein translation, cell-cycle progression and metabolism [8]. mTORC1 signalling senses extracellular nutrients and regulates response metabolic changes. For instance, insulin, growth factors and amino acids activate mTORC1 signalling via inhibiting tuberous sclerosis complex 1/2 (TSC1/2) under nutrient-enriched conditions [9]. By contrast, decrease in environmental oxygen and intracellular ATP, such as glucose depletion or hypoxia, may inhibit mTORC1 signalling at least partly through AMPK/tuberous sclerosis complex (TSC) or limited energy supply [10,11]. Once activated, mTORC1 directly phosphorylates S6K1, 4E-BP1 and a growing number of other downstream targets to develop anabolism whereas mTORC1 inactivation promotes anabolic events, such as lipid oxidation and autophagy [12].

One of the most important roles of mTORC1 on metabolism is to control lipid homoeostasis [12]. It has long been appreciated that mTORC1 controls the balance of lipid synthesis and oxidation. mTORC1 signalling promotes global expression of SREBP1 and SREBP2 targets and drives lipogenesis in response to both physiological and genetic stimulations [13]. Of note, mTORC1 signalling has been suggested to increase SREBP1 activation in a p70S6K1-dependent manner in many cell and animal models [14,15]. Besides lipid synthesis, mTORC1 also controls lipid oxidation. It has been proved that rapamycin dramatically inhibits mTORC1 activity and has profound effects on promoting β-oxidation as decreasing flux into anabolic storage pathways [16,17]. Thus, mTORC1 inhibition by rapamycin seems to induce a “fasted” phenotype, including decreased expression of lipogenesis genes and increased expression of lipid oxidation genes. Previous studies have reported that under hypoxia conditions, mTORC1 activity is dramatically inhibited [10]. Therefore, hypoxia cells must shift their metabolism to meet the energy demand. Although studies have demonstrated that mTORC1 may regulate hypoxia lipid metabolism in several cell types, the lipid metabolic shift in hypoxia lymphocytes have not been well defined. Again, clear mechanisms of how lipid metabolism alters according to oxygen levels in lymphocytes should be further elucidated.

Here, we report that mTORC1 signalling may be a central regulator of lipid metabolism in hypoxia lymphocytes. Under hypoxia conditions, lymphocytes can reduce cellular mTORC1 activity, which causes decreased lipid synthesis and increased lipid oxidation. By knockdown TSC1, a well-known mTORC1 inhibitor, mTORC1 activity is constitutively activated. The constitutively activated mTORC1 impairs the balance of lipid synthesis and oxidation and enhances hypoxia-induced cell apoptosis. Re-inactivation of mTORC1 activity via rapamycin may resist hypoxia-induced cell death in TSC1 knockdown lymphocytes. Our findings provide a deep insight into mTORC1 in the metabolic balance of lipid synthesis and oxidation, and imply that mTORC1 activity may be precisely regulated for the lipid homoeostasis in lymphocytes.

## Materials and methods

### Antibodies and reagents

In the Western blot assays, all the antibodies were of high quality. The pp70S6K, p70S6K, pS6, S6, p4EBP1, 4EBP1, TSC1, β-actin, pro- and cleaved-caspase-3 antibodies were purchased from Cell Signaling Technology (Danvers, MA, USA), which were widely used for mTORC1 signalling study. For cell culture, RPMI-1640 and FBS for mouse spleen lymphocyte culture were purchased from GIBCO Invitrogen (Carlsbad, CA, USA). Rapamycin was from Invitrogen (Carlsbad, CA, USA). Lentiviral shRNAs to mouse TSC1 were previously described [18]. The sequences of the oligonucleotides are as follows: TSC1 shRNA sense: 5′-CCGGGGGAGGTCAACGAGCTCTATTAAAGCTTTAATAGAGCTCGTTGACCTCCCTTTTTC-3′; TSC1 shRNA antisense: 5′-AATTGAAAAAGGGAGGTCAACGA GCTCTATTAAAGCTTTAATAGAGCTCGTTGACCT CCC-3′. Other chemicals were of the highest purity available.

### Cell culture preparations

Mouse lymphocyte cells were prepared from C57BL/6 mice. For mouse lymphocyte preparations, the spleens were collected and disrupted with a grinder in PBS (pH 7.4). After a 10-min centrifugation at 1500 rpm to separate debris, erythrocytes were lysed using ammonium chloride reagent. The cells were washed twice with PBS and suspended in 0.5 ml of cold RPMI-1640 medium with 10% FBS plus 1% antibiotics (penicillin and streptomycin). Spleen lymphocyte number was determined with a blood-cell counting chamber (Erma, Japan). The viability of lymphocytes was determined by Trypan Blue exclusion.

### Hypoxia and drug treatment

For hypoxia treatment, primary mouse lymphocytes were cultured under hypoxia (0.5% O_2_) conditions, while controls under normoxia (20% O_2_) for 24 h [6]. To elevate mTORC1 activity, lentiviral shRNAs to mouse TSC1 were transfected with these cells using Lipofectamine 2000. To inhibit mTORC1 activity, rapamycin (100 nM) was applied to normoxia and hypoxia lymphocytes for 24 h. For Western blots and real-time PCR experiments, lymphocyte cells were plated in six-well plates at 1.0 × 10^6^ cells/ml. After culturing, the cells were harvested for subsequent examinations.

### Lysates preparation and Western blots

To assay mTORC1 activity in primary lymphocytes, total proteins were extracted from cell harvest. For Western blots, prepared cells were trypsinized and harvested, washed with PBS once and resuspended in PBS buffer containing 1% Triton X-100 and protease/phosphatase inhibitors. After brief sonication, cell lysates were centrifuged at 13000 rpm for 5 min. Protein concentration was determined so that equivalent amounts of lysate based on protein concentration was added to an equal volume of Laemmli buffer and boiled for 10 min. For Western blot analysis, the procedure was performed according to standard protocol. Finally, proteins were detected by Super Signal^®^ ECL development (Thermo Scientific Pierce) reagent and exposed to films (Kodak). The protein level quantification was carried out by ImageJ.

### Quantitative real-time PCR

To assay gene expressions of lipogenesis and lipid oxidation in primary lymphocytes, total RNA was extracted from tissues using TRizol reagent (Invitrogen). RNA was subjected to reverse transcription with reverse transcriptase according to manufacturer’s instructions (Fermentas). Quantitative real-time PCR was performed using Bio–Rad iQ5 system, and relative gene expression was normalized to internal control as *β-actin*. Primer sequences for SYBR Green probes of target genes are shown in Table 1.

**Table 1:** Primer sequences for SYBR Green probes of target genes

Gene	Primer sequence (5′→3′)
*Srebp1c-F*	GGAGCC ATGGATTGCACATT
*Srebp1c-R*	GCTTCCAGAGAGGAGGCCAG
*Acc1* (acetyl-CoA carboxylase 1)*-F*	CCTCCGTCAGCTCAGATACA
*Acc1-R*	TTTACTAGGTGCAAGCCAGACA
*Fasn* (fatty acid synthase)*-F*	TGGGTTCTAGCCAGCAGAGT
*Fasn-R*	ACCACCAGAGACCGTTATGC
*Atgl* (adipose triacylglycerol lipase)*-F*	TGTGGCCTCATTCCTCCTAC
*Atgl-R*	TGCTGGATGTTGGTGGAGCT
*Pparα* (peroxisome proliferator activated receptor alpha)*-F*	TGTTTGTGGCTGCTATAATTTGC
*Pparα-R*	GCAACTTCTCAATGTAGCCTATGTTT
*Cpt1-α* (carnitine palmitoyltransferase 1-a)*-F*	GGAGAGAATTTCATCCACTTCCA
*Cpt1-α-R*	CTTCCCAAAGCGGTGTGAGT
*β-actin-F*	GAGACCTTCAACACCCCAGC
*β-actin-R*	ATGTCACGCACGATTTCCC

### Statistical analysis

Data represent the mean and S.E.M. ANOVA tests for comparisons were performed for all statistical significance analysis using GraphPad Prism software; *P<0.05, **P<0.01 and ***P<0.001.

## Results

### TSC1 knockdown rescues mTORC1 inactivation in hypoxia lymphocytes

**Figure 1:**
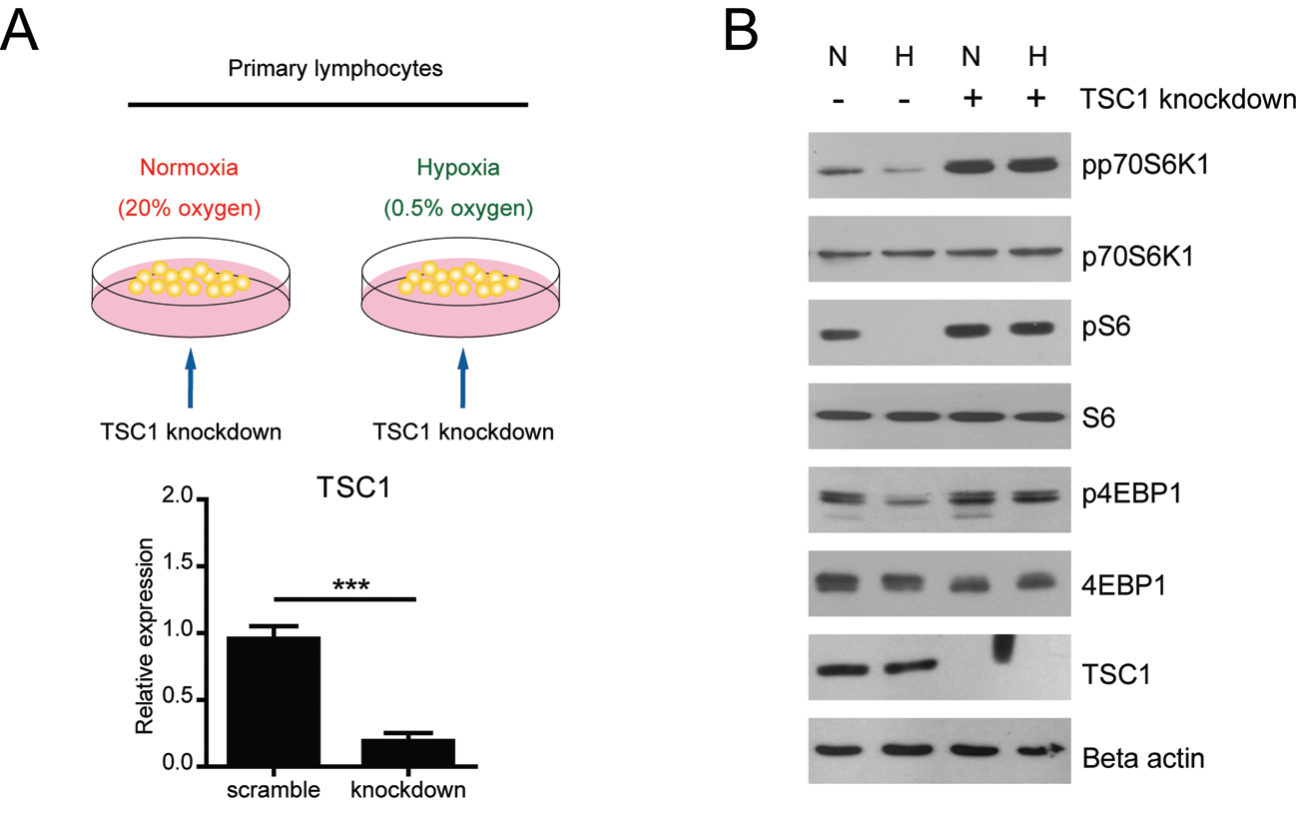
TSC1 knockdown rescues mTORC1-inactivation in hypoxia lymphocytes (**A**) A schematic model showing the experiment design on hypoxia mTORC1 signalling. Primary mouse spleen lymphocytes were cultured in normoxia (20% O_2_) and hypoxia (0.5% O_2_). To activate mTORC1 signalling, TSC1 knockdown was introduced to these lymphocytes. The knockdown efficiency of TSC1 was confirmed by real-time PCR. (**B**) Western blots showed that mTORC1 activity, indicated by phosphorylations of p70S6K, S6 and 4EBP1, was down-regulated in hypoxia and re-upregulated by TSC1 knockdown. Results are averages of three independent experiments.

It has long been implicated that mTORC1 signaling maintains metabolic homeostasis in various types of cells under different conditions. [17]. For example, hypoxia induces changes in lipid metabolism via mTORC1 signalling [19,20]. However, how mTORC1 signalling is precisely regulated under hypoxia in lymphocytes is still not well defined. Thus, we first isolated and cultured mouse primary spleen lymphocytes in normoxia and hypoxia (Figure 1A). Then, we examined mTORC1 activity in normoxia and hypoxia lymphocytes. Biochemical results showed that protein levels of markers of mTORC1 signalling (pp70S6K, pS6 and p4EBP1) were all decreased in hypoxia, which was consistent with the previous report [20] (Figure 1B). To further manipulate mTORC1 activity, we knockdown TSC1 gene, which is a well-defined upstream inhibitor of mTORC1 activity [21]. We noted that the knockdown efficiency is very high in these cells, because TSC1 expression was dramatically inhibited in both mRNA and protein levels (Figure 1A and B). Subsequently, we found that mTORC1 activity (indicated by pp70S6K, pS6 and p4EBP1) was obviously increased in both normoxia and hypoxia (Figure 1B). All these results suggest hypoxia reduces mTORC1 activity in lymphocytes and TSC1 knockdown may constitutively activate mTORC1 in hypoxia.

### Hypoxia shifts lipogenesis to lipid oxidation via mTORC1 signalling in lymphocytes

**Figure 2:**
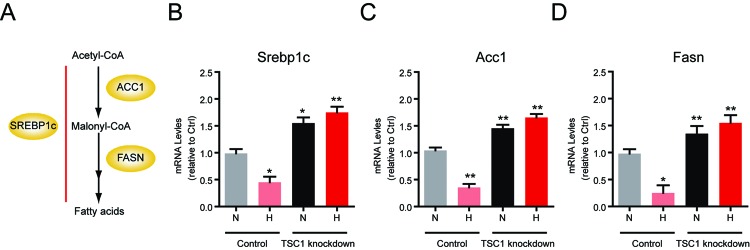
Hypoxia decreases lipogenesis via mTORC1 signalling in lymphocytes (**A**) A schematic model showing the signalling of lipogenesis. *Srebp1c* controls expressions of lipogenesis genes, such as *Acc1* and *Fasn*, downstream of mTORC1 signalling. (**B–D**) Real-time PCR results showed that mRNA levels of lipogenesis genes, including *Srebp1c* (B), *Acc1* (C) and Fasn (D) were decreased in hypoxia. TSC1 knockdown increases these gene expressions in both normoxia and hypoxia. Results are averages of three independent experiments. Data represent mean ± S.E.M.; *P<0.05 and **P<0.01.

The most biological function of mTORC1 signaling is to maintain metabolic homeostasis in various types of cells, such as lipid homoeostasis.. mTORC1 is a master regulator of lipid *de novo* synthesis (lipogenesis) and promotes anabolism [12]. Thus, we first investigated whether hypoxia-induced mTORC1 inactivation would like to decrease lipogenesis in lymphocytes. The expression of lipogenesis enzymes is controlled by the SREBP1 transcription factor [22] (Figure 2A). By real-time PCR assays, we found that expressions of lipogenesis genes, in particular *Srebp1c, Acc1* and *Fasn*, were dramatically reduced by mTORC1 inactivation in hypoxia (Figure 2B–D). We also noted that TSC1 knockdown could constitutively activate mTORC1 in hypoxia and thus lead to the up-regulation of lipogenesis genes (*Srebp1c, Acc1* and *Fasn*) in both normoxia and hypoxia.

**Figure 3:**
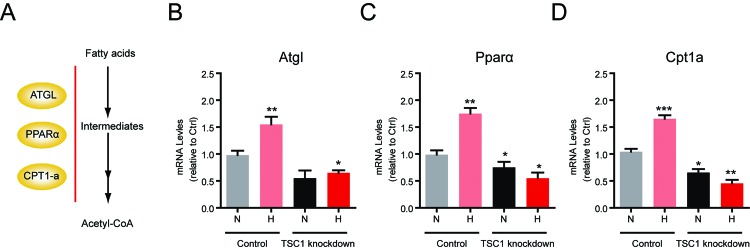
Hypoxia increases lipid oxidation via mTORC1 signalling in lymphocytes (**A**) A schematic model showing the signalling of lipid oxidation. *Atgl, Pparα* and *Cpt1-α* are important genes involved in lipid β-oxidation. (**B–D**) Real-time PCR results showed that mRNA levels of lipid oxidation genes, including *Atgl* (B), *Pparα* (C) and *Cpt1-α* (D) were increased in hypoxia. TSC1 knockdown decreases these gene expressions in both normoxia and hypoxia. Results are averages of three independent experiments. Data represent mean ± S.E.M.; *P<0.05, **P<0.01 and ***P<0.001.

On the other hand, mTORC1 has been proved to regulate lipid oxidation, which is a typical catabolism to provide energy source under hypoxia [16] (Figure 3A). We next study whether hypoxia-induced mTORC1 inactivation would like to increase lipid oxidation in lymphocytes. It is found that expressions of lipid oxidation genes, such as *Atgl, Pparα* and *Cpt1-*α, were increased by mTORC1 inactivation in hypoxia (Figure 3B–D). Consistently, we found that TSC1 knockdown could resist hypoxia-induced lipid oxidation gene expression, due to mTORC1 reactivation. Taken together, all of our findings indicate that hypoxia may alter lymphocyte lipid metabolism by shifting lipogenesis to lipid oxidation.

### TSC1 knockdown enhances hypoxia-mediated lymphocyte death via mTORC1 activation

**Figure 4:**
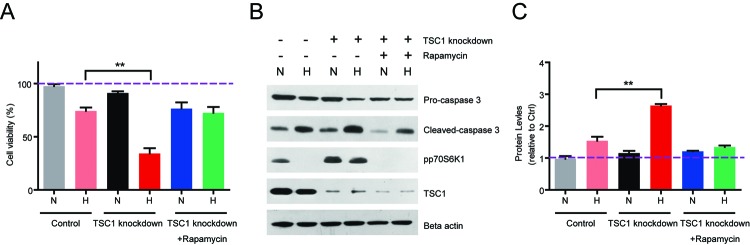
TSC1 knockdown enhances hypoxia-mediated lymphocyte death via mTORC1 activation (**A**) MTT assay showed that hypoxia reduces cell viability in hypoxia lymphocytes, whereas TSC1 knockdown enhances the cell death. Rapamycin treatment may rescue TSC1 knockdown-mediated cell death in hypoxia lymphocytes. (**B–C**) Western blots and quantifications showed that hypoxia induces cleaved caspase-3 protein levels and TSC1 knockdown enhances it. Rapamycin treatment may rescue TSC1 knockdown-induced cleaved caspase-3 in hypoxia lymphocytes. Results are averages of three independent experiments. Data represent mean ± S.E.M.; **P<0.01.

Since TSC1 knockdown could activate mTORC1 pathway and alter lipid metabolism in hypoxia, we propose that altered lipid metabolism may cause cell death in hypoxia lymphocytes. Thus, we examined the cell viability in both control and TSC1 knockdown hypoxia lymphocytes. The MTT assay results showed that hypoxia reduces cell viability in hypoxia lymphocytes, whereas TSC1 knockdown enhances the cell death (Figure 4A). Consistently, biochemical results confirmed that hypoxia increases the protein level of cleaved caspase-3, which is a well-known apoptosis marker. And TSC1 knockdown dramatically increased the protein level of cleaved caspase-3 compared with its control counterparts (Figure 4B and C).

Next, we assumed that TSC1 knockdown-induced lymphocyte death may be mediated by mTORC1 pathway. To test this hypothesis, we applied mTORC1 inhibitor rapamycin to block mTORC1 activity and investigated the cell viability. Results showed that rapamycin treatment could effectively block mTORC1 activity in both control and TSC1 knockdown lymphocytes (Figure 4B). Accordingly, the cell viability was increased by rapamycin treatment compared with TSC1 knockdown in hypoxia (Figure 4A). Finally, we found that the protein level of cleaved caspase-3 was decreased by rapamycin treatment compared with TSC1 knockdown counterparts (Figure 4B and C). Based on the above results, our findings suggest that ectopic activation of mTORC1 pathway may enhance cell death in hypoxia lymphocytes.

## Discussion

**Figure 5:**
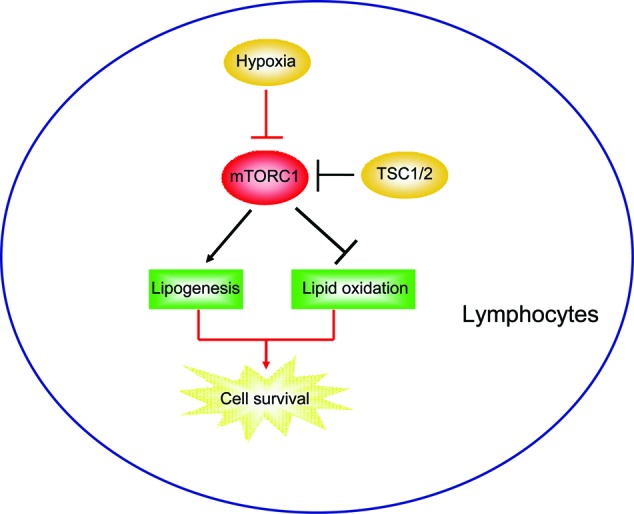
Model Schematic representation explaining the network of mTORC1 signalling on hypoxia lipid metabolic shift from lipogenesis to lipid oxidation. Hypoxia reduces lymphocyte mTORC1 activity and shifts lipid synthesis to lipid oxidation whereas knockdown TSC1 constitutively activates mTORC1 activity and impairs the hypoxia-induced metabolic shift.

Regulation of lipid metabolism has emerged as an important aspect of lymphocyte cell metabolism [23]. The balance of lipid synthesis and oxidation should be precisely modulated under special conditions, such as hypoxia [24]. Hypoxia reduces the availability of glucose-derived carbons for lipid synthesis and can activate lipid oxidation to meet the energy demand [19]. However, mechanisms of how lipid metabolism alters according to oxygen levels in lymphocytes are not well defined. In the present study, we identified that mTORC1 signalling plays a vital role in the balancing of lipid synthesis and oxidation in hypoxia lymphocytes. We found that hypoxia reduces lymphocyte mTORC1 activity and shifts lipid synthesis to lipid oxidation. However, knockdown TSC1 constitutively activates mTORC1 activity and impairs the hypoxia-induced metabolic shift. Thus, TSC1 knockdown enhances hypoxia-induced cell apoptosis. Re-inactivation of mTORC1 activity via rapamycin in TSC1 knockdown lymphocytes may help in cell survival in hypoxia. Our findings indicate that mTORC1 activity may be precisely regulated in hypoxia lymphocyte for the lipid homoeostasis and cell survival (Figure 5).

Hypoxia is a common feature of low environmental oxygen levels and alters cellular lipid metabolism on several levels [25]. Hypoxia reduces the availability of glucose-derived carbons for lipid synthesis, which may save energy for other metabolic events. On the other hand, hypoxia may promote lipid oxidation to utilize fatty acids for energy production. Here, we proved that all of these lipid metabolic alterations may be regulated by mTORC1 signalling. We found that like many other hypoxia models, mTORC1 activity was down-regulated in low oxygen conditions. Thus, mTORC1-mediated lipogenesis and lipid oxidation were metabolically altered. Therefore, mTORC1 activity should be naturally reduced for cell survival in hypoxia.

mTORC1 signalling is a master regulator of many anabolic processes [26]. For example, mTORC1 positively promotes protein synthesis, which is an energy-consuming event [27]. As for *de novo* lipogenesis, acetyl-CoA was converted into fatty acids by the addition of two-carbon units. Through lipogenesis, the energy can be efficiently stored in the form of fatty acids. mTORC1 is the central regulator of lipogenesis [28]. Inhibition of mTORC1 blocks expressions of genes involved in lipogenesis and impairs the nuclear accumulation of the SREBPs [29]. Moreover, mTORC1 phosphorylates CRTC2 and attenuates its inhibitory effect on COPII-dependent SREBP1 maturation and downstream lipogenesis [30]. Under hypoxia conditions, low oxygen dramatically reduces oxidative phosphorylation and ATP production [31]. Thus, cells must adapt to metabolic alterations to save the energy and utilize stored energy.

Here, we showed that in hypoxia, lymphocyte reduces mTORC1 activity to down-regulate energy-consuming lipogenesis and up-regulate energy using lipid oxidation. We found that cell viability was decreased by mTORC1 activation and enhanced lipogenesis in hypoxia. We speculate that the limited energy supply may be partly responsible for this finding. mTORC1 is a central regulator of anabolism, such as protein synthesis and lipogenesis. However, these procedures are highly energy consuming. Under hypoxia conditions, cells develop catabolic procedures, such as autophagy, to save the energy for cell survival. Therefore, excessive energy consumption may promote cell death in hypoxia, whereas TSC1 knockdown activates mTORC1 signalling and disrupts the shift of lipid metabolism. Thus, mTORC1 activated lymphocyte may lack energy supply for survival in hypoxia and finally step to apoptosis. Therefore, mTORC1 signalling acts as a key factor in the balance of lipid anabolism and catabolism, especially in metabolically altered conditions (such as hypoxia).

In summary, the present findings supported the fact that mTORC1 signalling may be a central regulator of lipid homoeostasis in lymphocytes. Under hypoxia condition, mTORC1 activity is reduced and shifts lipid synthesis to lipid oxidation. However, knockdown TSC1 constitutively activates mTORC1 activity and impairs the hypoxia-induced metabolic shift. Therefore, TSC1 knockdown may enhance hypoxia-induced cell apoptosis. Re-inactivation of mTORC1 activity via rapamycin may resist hypoxia-induced cell death in TSC1 knockdown lymphocytes. Our findings indicated that mTORC1 activity might be precisely regulated in hypoxia lymphocyte for the lipid homoeostasis and cell survival.

## Abbreviations

4E-BP1: 4E-binding protein 1
Acc1: acetyl-CoA carboxylase 1
AMPK: AMP-activated protein kinase
Atgl: adipose triacylglycerol lipase
COPII: coat protein complex II
CPT1-α: carnitine palmitoyltransferase 1-a
CRTC2: CREB regulated transcription coactivator 2
Fasn: fatty acid synthase
PPARα: peroxisome proliferator activated receptor alpha
p70S6K1: p70 ribosomal protein S6 kinase 1
pp70S6k: phospho-p70 ribosomal protein S6 kinase
p4EBP1: phospho-4E-binding protein 1
pS6: phospho-ribosomal protein S6
mLST8: SEC13 protein 8
mTOR: mammalian target of rapamycin
mTORC1: mTOR complex 1
RPMI-1640: roswell Park Memorial Institute-1640
S6K1: ribosomal protein S6 kinase 1
SREBP1c: sterol regulatory element-binding protein 1c
S6: ribosomal protein S6
TSC: tuberous sclerosis complex
TSC1: tuberous sclerosis complex 1
